# Genome-Wide Identification and Transferability of Microsatellite Markers between Palmae Species

**DOI:** 10.3389/fpls.2016.01578

**Published:** 2016-10-25

**Authors:** Yong Xiao, Wei Xia, Jianwei Ma, Annaliese S. Mason, Haikuo Fan, Peng Shi, Xintao Lei, Zilong Ma, Ming Peng

**Affiliations:** ^1^Hainan Key Laboratory of Tropical Oil Crops Biology/Coconut Research Institute, Chinese Academy of Tropical Agricultural SciencesWenchang, China; ^2^College of Agriculture, Hainan UniversityHaikou, China; ^3^Department of Plant Breeding, IFZ Research Centre for Biosystems, Land Use and Nutrition, Justus Liebig University GiessenGiessen, Germany; ^4^Institute of Tropical Bioscience and Biotechnology, Chinese Academy of Tropical Agricultural ScienceHaikou, China

**Keywords:** palm, *Elaeis guineensis*, *Phoenix dactylifera*, microsatellite, cross-genera transferability

## Abstract

The Palmae family contains 202 genera and approximately 2800 species. Except for *Elaeis guineensis* and *Phoenix dactylifera*, almost no genetic and genomic information is available for Palmae species. Therefore, this is an obstacle to the conservation and genetic assessment of Palmae species, especially those that are currently endangered. The study was performed to develop a large number of microsatellite markers which can be used for genetic analysis in different Palmae species. Based on the assembled genome of *E. guineensis* and *P. dactylifera*, a total of 814 383 and 371 629 microsatellites were identified. Among these microsatellites identified in *E. guineensis*, 734 509 primer pairs could be designed from the flanking sequences of these microsatellites. The majority (618 762) of these designed primer pairs had *in silico* products in the genome of *E. guineensis*. These 618 762 primer pairs were subsequently used to *in silico* amplify the genome of *P. dactylifera*. A total of 7 265 conserved microsatellites were identified between *E. guineensis* and *P. dactylifera*. One hundred and thirty-five primer pairs flanking the conserved SSRs were stochastically selected and validated to have high cross-genera transferability, varying from 16.7 to 93.3% with an average of 73.7%. These genome-wide conserved microsatellite markers will provide a useful tool for genetic assessment and conservation of different Palmae species in the future.

## Introduction

Palmae is a monocot family containing 202 genera and approximately 2800 species mainly distributed in tropical and subtropical regions. Palm trees generally have a solitary shoot bearing a crop of very large leaves. The majority of palm trees are used as landscape plants, planted along both sides of the street. However, there are also some important economic crops in this family, such as *Elaeis guineensis, Phoenix dactylifera, Cocos nucifera* and *Areca catechu*. Of the four economic crops, the African oil palm *E. guineensis* has the largest plantation area (17 million hectares), producing 50 million tons of palm oil annually. Due to its economic importance, the whole genome sequence of *E. guineensis* has already been completed and was released in August 2013 (Singh et al., 2013). The draft genome sequence of related species *P. dactylifera* has also been reported (AI-Dous et al., 2011; AI-Mssallem et al., 2013). The genome sizes of *E. guineensis* and *P. dactylifera* are 1.535 and 605 Mb, respectively. However, only 657.86 Mb (42.86%) can be mapped into the 16 assembled chromosomes of *E. guineensis*, and some chromosomes are not assembled in *P. dactylifera*. Previous research based on phylogenetic dating has shown a divergence 65 Mya between *E. guineensis* and *P. dactylifera* (Singh et al., 2013). Except for these species, other Palmae are still under-studied and few genetic and genomic resources are available. This is an obstacle to the conservation and genetic assessment of Palmae species, especially those that are currently endangered.

Microsatellites (simple sequence repeats, SSRs) are tandem DNA repeats which are mostly distributed throughout non-coding regions of eukaryotic genomes. Microsatellite markers are generally co-dominant and high polymorphic, and are widely used for constructing genetic maps, diversity analysis and quantitative trait locus mapping (Qiu et al., 2006; Xiao et al., 2010; Zhang et al., 2012; Xia et al., 2014). Due to lack of selection pressure on these non-coding sequences, microsatellites are generally regarded as neutral genetic markers. Recently, microsatellites have been also validated to have important biological functions, such as the regulation of chromatin organization, DNA metabolic processes, gene activity and RNA structure (Li et al., 2002, 2004; Gemayel et al., 2010). Some research has already been done to identify microsatellite markers via probe hybridization against genomic/cDNA clones (Rivera et al., 1999). This method of developing microsatellites is still low efficiency and high cost. With the use of next-generation sequencing (NGS) technology, it became possible to develop large numbers of molecular markers through *in silico* analysis of available bacterial artificial chromosome (BAC) sequences (Xu et al., 2010), BAC-end sequences (BES) (Bohra et al., 2011), genome survey sequences (WGSs) (Marek et al., 2001), expressed sequence tags (Low et al., 2008), and whole genome shotgun sequences (WGSs) (Shi et al., 2013). In the Palmae family, cDNA library tags and transcriptome data have been used to develop EST-SSRs in *E. guineensis* (Xiao et al., 2014), *P. dactylifera* (Zhao et al., 2012), and *C. nucifera* (Xiao et al., 2014). With the release of the whole genome sequences of *E. guineensis* and *P. dactylifera*, this will provide an opportunity to develop larger number of microsatellite markers and ascertain their distribution across different chromosomes in the two Palmae species. However, except for these two Palmae species, identification of microsatellite markers is difficult because of the lack or scarcity of available sequence data in other Palmae species.

It has been well recognized that some molecular markers (including microsatellite markers) can be transferable from different genotypes within or between species or even between genera (Wang et al., 2012). Such interspecific or intergeneric transferability for markers will provide some useful genetic information for poorly-studied related species, contributing to conservation, genetic assessment, and construction of linkage maps. Meanwhile, the transferability of microsatellite markers depends on the genetic relationship between the species. Mnejja et al. (2010) reported that microsatellite markers developed from peach and almond had a higher transferability rate to other *Prunus* species than to three other non-*Prunus* rosaceous genera (Mnejja et al., 2005). In previous research, microsatellite cross-species transferability is identified generally via PCR amplification in different related species using primer pairs designed from one species (Decroocq et al., 2003; Gasic et al., 2009). This method to identify cross-species transferability of primer pairs is very low efficiency. Meanwhile, the number of primer pairs with cross-species transferability identified is also limited.

Among the Palmae family, the whole genome sequences of *E. guineensis* and *P. dactylifera* have been completed and released. This study (1) identified a large number of microsatellites and analyzed the frequency and distribution of microsatellites in the two palm species, respectively; (2) analyzed the distribution of these microsatellites in 16 chromosomes of *E. guineensis*; (3) identified conversed microsatellites genome-wide between *E. guineensis* and *P. dactylifera*; (4) validated the cross-genera transferability of these conserved microsatellites in Palmae.

## Materials and methods

### Plant materials and DNA extraction

Thirty-two samples from different palm species were collected from the Palmae germplasm nursery of the Coconut Research Institute in Wenchang, Hainan, China: *Chrysalldocarpus lutescens, Livistona australis, Dictyosperma Album, Cary mitis, Corypha umbraculifea, Latanic lontaroides, Phoenix loureirii, Cyrtostachys renda, Veitchia merrillii, Hyophorbe verschaffeltii, Chrysalidocarpus lucubensis, Sabal palmetto, Wodyetia bifurcate, Trachycarpus nana, Areca triandra, Rhapis excels, Butia capitata, Cryosophila albida, Chamaedorea Elegans, Pritchardia pacifica, Dypsis decaryi, Phoenix robusta, Arenga engieri, Borassus flabellifer, Bismarckia Hildebr, C. nucifera, E. guineensis, A. catechu, Chamaerops ritchieana*, Iraq candy date, *P. dactylifera*, and *Hyophorbe lagenicaulis*. Detailed information for these individual species is listed in Figure S1. DNA samples were prepared from young leaves of the 32 palm species samples using the mini-CTAB method (Stewart and Via, 1993).

### Sources of genome sequences and gene annotation information

The genomic sequences from *E. guineensis* and *P. dactylifera* were downloaded from the National Center of Biotechnology Information (NCBI). A total of 1056.95, 1038.22, and 506.67 Mb from 202 467, 219 249, and 143 380 contigs were used for microsatellite identification in *E. guineensis* and *P. dactylifera*, respectively.

A total of 30 057 genes were predicted by the National Center for Biotechnology information in *E. guineensis*. The gene annotation information was downloaded from NCBI. The mining of transposable elements (TEs) in *E. guineensis* genome was done using RepeatMasker (Smit et al., 2013/2015) software with the TE database of *Arabidopsis* and rice.

### Identification of putative SSRs and primer design

The SSR analysis software Msatfinder (https://github.com/knirirr/Msatfinder) was used to identify all possible mono, di-, tri-, tetra, penta- ,and hexa-nucleotide SSRs with a minimum set of 12, 4, 4, 4, 4, and 4 repeats, respectively (Thurston and Field, 2005). Subsequently, primer pairs were designed based on the sequences flanking the SSRs using Primer 3 software (Rozen and Skaletsky, 1999), with melting temperatures 58–62°C, primer lengths 18–24 bp, GC content 45–55%, expected fragment size 100–250 bp.

### ePCR

A total of 734 509 primer pairs were designed based on the flanking sequences of 814 383 microsatellites of *E. guineensis* with primer 3. These primer pairs were used to *in silico* amplify the genomic sequences of *P. dactylifera* using the e-PCR software (www.ncbi.nlm.nih.gov/tools/epcr) (Schuler, 1997; Rotmistrovsky et al., 2004). The primer nucleotide mismatch allowed was no more than 1 nt. Meanwhile, the size difference allowed between the PCR products was less than 100 bp between the *in silico* PCR product in *P. dactylifera* and the expected PCR product in *E. guineensis*.

### PCR amplification and electrophoresis

PCR amplifications were performed in 10 ul reaction mixtures containing 100 ng genomic DNA, 1 × PCR buffer, 2 mM MgCl_2_, 1 U Taq DNA polymerase (TakaRa, China), 0.5 uM of each primer and 0.2 mM dNTP mix, with the following program: denaturation for 4 min at 94°C, 30 cycles of 30 s at 94°C, 30 s at 56°C and 30 s at 72°C for elongation, with a final extension of 7 min at 72°C. PCR products were electrophoretically separated on 1% agarose gels. Product sizes were determined by comparison with a 100 bp DNA ladder.

## Results

### Frequency and distribution of simple sequence repeats in *Elaeis guineensis* and *Phoenix dactylifera*

A total of 814 383 and 371 629 mononucleotide to hexanucleotide repeat microsatellite sequences were identified from 1057 and 507 Mb of assembled genomic sequences of *E. guineensis* and *P. dactylifera*, respectively (Table 1), with an average of 770.4 and 733 microsatellites per Mb, or one microsatellite per 1.29 and 1.36 Kb.

**Table 1:** **Number, repeat number and total length of the mononucleotide to hexanucleotide motifs of microsatellites in the assembled genomic sequences of ***Elaeis guineensis*** and ***Phoenix dactylifera*****.

**Motif**	***Elaeis guineensis***			***Phoenix dactylifera***		
	**Number**	**Repeat number (average)**	**Total length**	**Number**	**Repeat number**	**Total length**
Mono	78 184 (9.6%)	12–51 (13.632)	1 065 826	35 865 (9.7%)	12–115 (13.509)	484 504
a/t	74 526 (9.2%)	12–51 (13.634)	1 016 054	31 302 (8.4%)	12–80 (13.422)	420 148
c/g	3 658 (0.4%)	12–27 (13.606)	49 772	4 563 (1.2%)	12–115 (14.104)	64 356
Di	623 412 (76.6%)	4–84 (5.145)	6 415 480	270 256 (72.7%)	4–86 (5.491)	2 967 704
ca/tg	54 064 (6.6%)	4–30 (4.723)	510 666	22 687 (6.1%)	4–38 (4.985)	226 208
At	148 174 (18.2%)	4–80 (5.373)	1 592 412	55 616 (15%)	4–86 (5.385)	598 970
tc/ga	138 269 (17%)	4–62 (4.786)	1 323 526	65 817 (17.7%)	4–64 (5.417)	713 012
ct/ag	151 296 (18.6%)	4–40 (4.964)	1 502 004	65 900 (17.7%)	4–86 (5.741)	756 656
Ta	103 327 (12.7%)	4–84 (5.711)	1 180 136	42 285 (11.4%)	4–53 (5.661)	478 768
gt/ac	24 828 (3%)	4–31 (5.534)	274 794	13 993 (3.8%)	4–43 (5.664)	158 526
Cg	1 667 (0.2%)	4–14 (4.587)	15 294	2 034 (0.5%)	4–19 (4.393)	17 872
Gc	1 787 (0.2%)	4–12 (4.658)	16 648	1 924 (0.5%)	4–14 (4.598)	17 692
Tri	84 893 (10.4%)	4–81 (4.649)	1 184 076	49 147 (13.2%)	4–97 (4.85)	715 137
tct/aga	13 187 (1.6%)	4–22 (4.297)	170 001	4 074 (1.1%)	4–15 (4.765)	58 242
aag/ctt	10 901 (1.3%)	4–23 (4.576)	149 652	4 995 (1.3%)	4–26 (4.826)	72 312
ttc/gaa	9 462 (1.2%)	4–21 (4.521)	128 325	5 545 (1.5%)	4–21 (4.678)	77 823
att/aat	7 547 (0.9%)	4–55 (5.427)	122874	5 545 (1.5%)	4–95 (5.569)	69 597
tta/taa	5 627 (0.7%)	4–68 (5.304)	89 538	3 183 (0.9%)	4–50 (5.454)	52 083
ctc/gag	4 784 (0.6%)	4–16 (4.438)	63 693	3 635 (1%)	4–16 (4.692)	51 171
gga/tcc	3 843 (0.5%)	4–13 (4.491)	51 777	2 733 (0.7%)	4–14 (4.643)	38 070
Others	29 542 (3.6%)	4–84 (4.606)	408 216	19 437 (5.2%)	4–97 (4.737)	295 839
Tetra	18 828 (2.3%)	4–153 (4.716)	355 192	12 055 (3.2%)	4–96 (4.778)	230 372
ttta	1 014 (0.1%)	4–13 (4.613)	18 712	845 (0.2%)	4–12 (4.801)	16 228
tatg	1 018 (0.1%)	4–16 (5.031)	20 488	491 (0.1%)	4–15 (5.096)	10 008
aaat	1 550 (0.2%)	4–11 (4.567)	28 316	954 (0.3%)	4–10 (4.754)	18 140
aaag	659 (0.1%)	4–10 (4.423)	11 660	452 (0.1%)	4–15 (4.746)	8 580
aata	457 (0.1%)	4–12 (4.65)	8 500	491 (0.1%)	4–12 (4.707)	9 244
Others	14 130 (1.7%)	4–153 (4.733)	267 516	8 822 (2.4%)	4–96 (4.766)	168 172
Penta	6 690 (0.8%)	4–17 (4.379)	146 465	3 055 (0.8%)	4–72 (4.372)	66 780
aaaag	356 (0.04%)	4–10 (4.306)	7 665	173 (0.05%)	4–72 (4.726)	4 085
aaaat	410 (0.1%)	4–8 (4.324)	8 865	168 (0.05%)	4–8 (4.429)	3 720
Tttct	194 (0.02%)	4–7 (4.201)	4 075	79 (0.02%)	4–10 (4.646)	1 835
Tttta	252 (0.03%)	4–7 (4.29)	5 405	88 (0.02%)	4–8 (4.273)	1 880
Others	5 478 (0.7%)	4–17 (4.398)	120 455	2547 (0.7%)	4–23 (4.339)	55 260
Hexa	2 376 (0.3%)	4–41 (4.37)	62 304	1 251 (0.3%)	4–10 (4.315)	32 388

**Figure 1:**
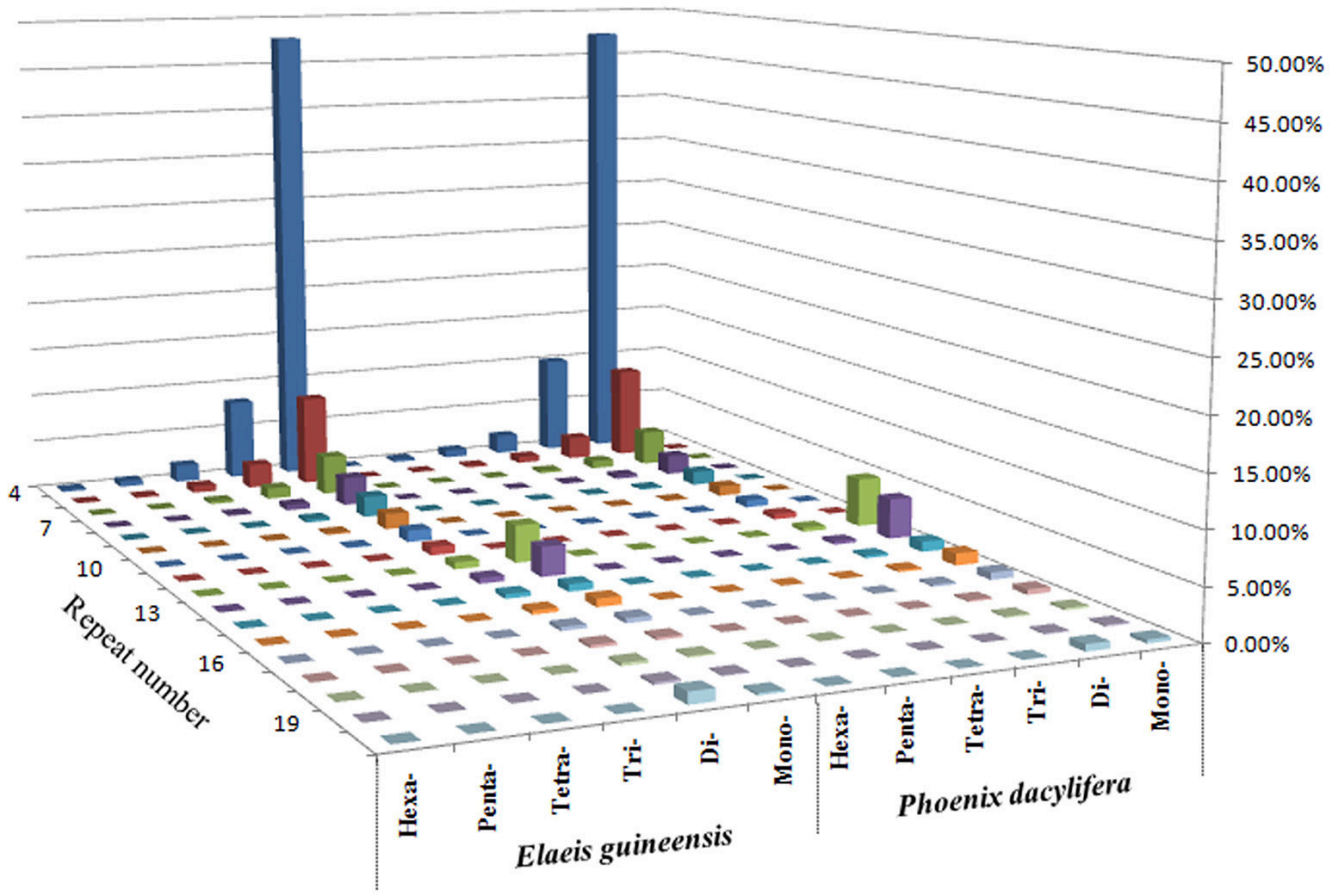
**Distribution with respect to the motif repeats of mono to hexa-nucleotide microsatellites based on the assembled genomic sequences of ***Elaeis guineensis*** and ***Phoenix dactylifera*****.

**Figure 2:**
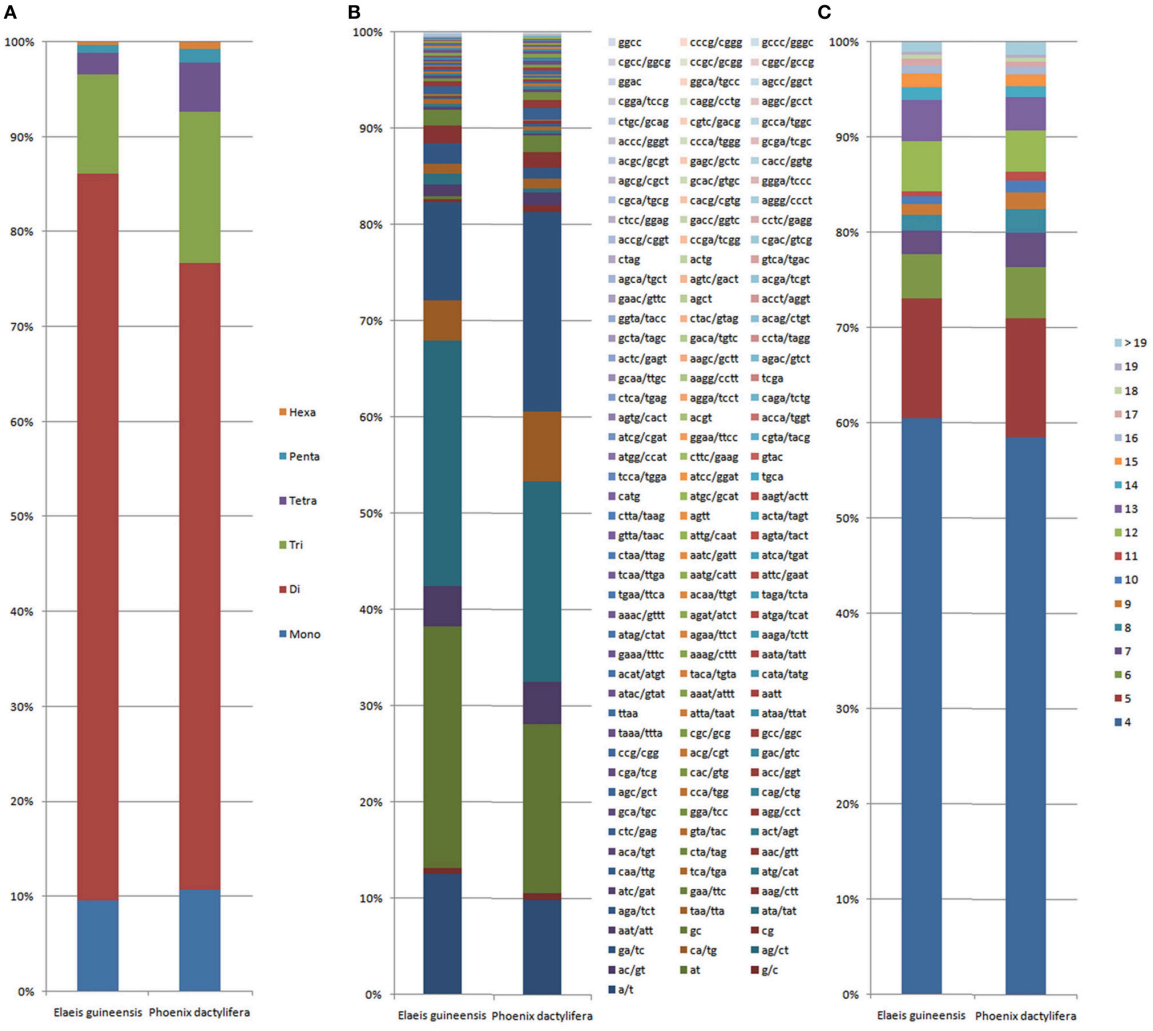
**Distribution with respect to the motif length (A), motif types (B) and repeat number (C) of microsatellites in the assembled genomes of ***Elaeis guineensis*** and ***Phoenix dactylifera*****. The vertical axis represents the percentage of SSRs with different motif lengths, motif types and repeat numbers.

Comparative analysis showed that the motif frequencies of microsatellites in the assembled genomic sequences of *E. guineensis* and *P. dactylifera* were almost identical (Figure 1). Among these identified microsatellites, dinucleotide motif types were the most abundant, comprising 76.6 and 72.7% of total motifs in *E. guineensis* and *P. dactylifera*. Trinucleotide motifs comprised the next largest proportion (10.4 and 13.2% in *E. guineensis* and *P. dactylifera* respectively), followed by mononucleotide motifs (9.6 and 9.7%). Smaller frequencies of tetranucleotide (2.3 and 3.2%), pentanucleotide (0.8 and 0.8%), and hexanucleotide (0.3 and 0.3%) motifs were also observed. The dominant motif types were all A/T in the two palm species, accounting for 95.3 and 93.2% of the total mononucleotide repeats, respectively. However, for dinucleotide motifs, the dominant/major motif was “AT” in *E. guineensis*, accounting for 23.8% of the total dinucleotide repeats, while the dominant/major motif was CT/AG in *P. dactylifera*, accounting for 24.4% of the total dinucleotide repeats. The trinucleotide motifs were relatively diverse. Among them, TCT/AGA was the richest repeat type in the tri-motifs of *E. guineensis*, accounting for 15.5% of total trinucleotide motifs in the species. However, in *P. dactylifera*, TTC/GAA and TTA/TAA were the dominant motifs, accounting for 11.3% of total trinucleotide motifs. Comparative analysis of motif type showed that the dominant motifs were all A/T rich, whereas the absent/scarce motifs were mostly C/G rich, which is in accordance with previous research results from *Brassica* species (Shi et al., 2013, Figure 2). Meanwhile, the distributions with respect to the motif repeat number of microsatellites in the assembled genome of the two palm species were almost identical. With increasing motif repeat number, the number of microsatellites with that motif significantly decreased (Figure 2).

### Genomic distribution

**Figure 3:**
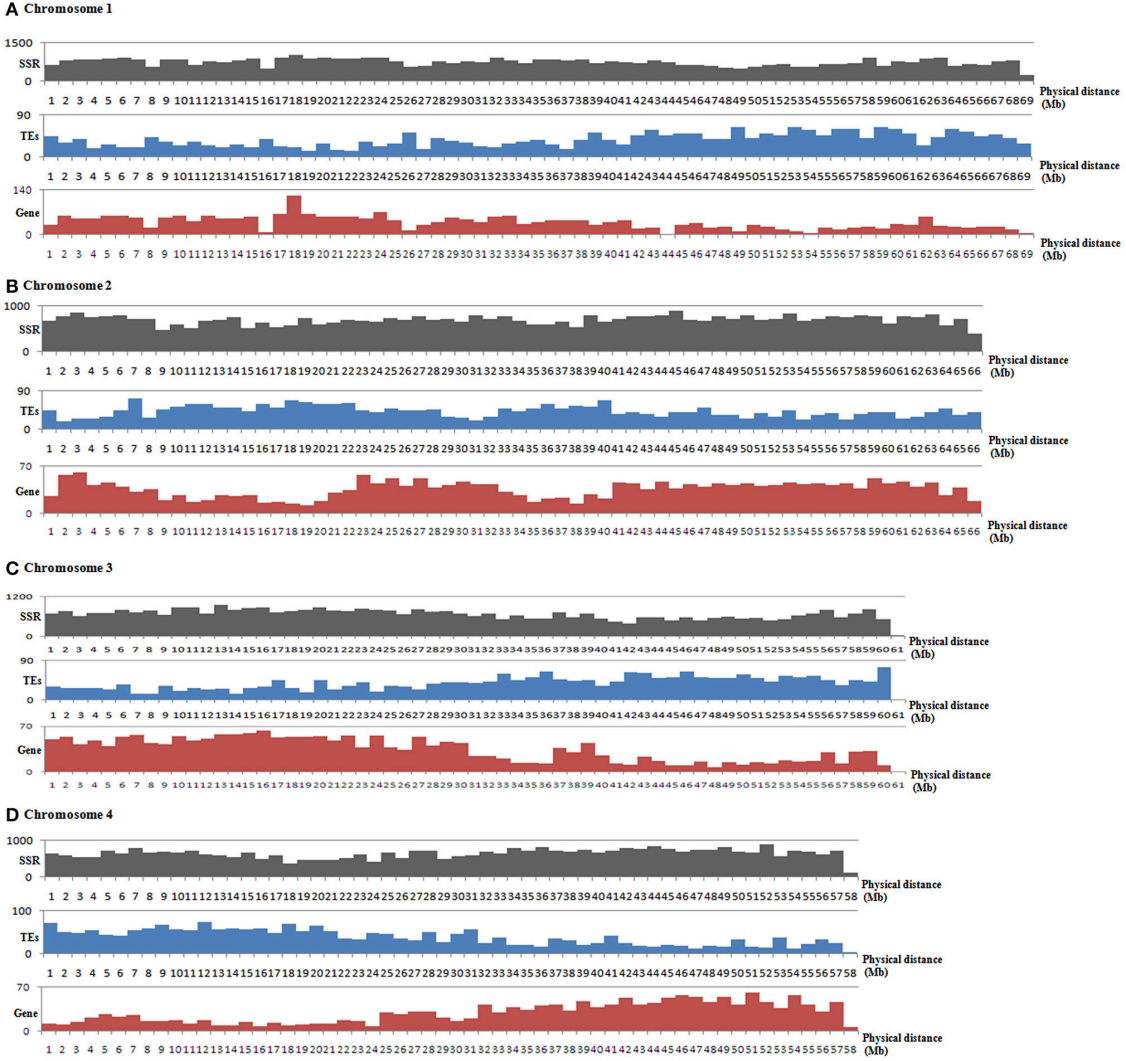
**Distributions and density of microsatellites as well as genes and TEs in the assembled chromosome 1 (A), chromosome 2 (B), chromosome (C), and chromosome 4 (D) of ***Elaeis guineensis*****. The frequency distributions of microsatellites, genes and TEs of other chromosomes are shown in Figure S2. The black, blue and red bars represent the number of microsatellites, TEs and genes, respectively. The horizontal axis shows the physical length of the assembled chromosomes.

The genome-wide distributions of microsatellites and their relation with the annotated genome components (including genes and TEs) were investigated based on the assembled genomic sequences of *E. guineensis*. A total of 814 383 microsatellites were identified in the assembled sequences of *E. guineensis*. Among these identified microsatellites, only 447 244 (54.92%) could be mapped to the 16 chromosomes of *E. guineensis*. The distribution of the mapped microsatellites across the different chromosomes was uneven (Table 2). A higher microsatellite density was found in chromosome 1 (723.71 microsatellites per Mb), chromosome 2 (707.23 microsatellites per Mb), chromosome 10 (724.4 microsatellites per Mb), and chromosome 19 (735.56 microsatellites per Mb) compared to the other chromosomes. Gene density was also comparatively high in these four chromosomes. Meanwhile, the frequency of microsatellites was high near both ends but low near the middle of all the chromosomes (Figure 3 and Figure S2): microsatellites had high density in chromosome peri-telomeres. The distribution of microsatellites was highly correlated with the distribution of annotated genes, but negatively correlated with the distribution of annotated TEs (Figure 3 and Table 2).

**Table 2:** **The density of microsatellites, genes and transposable elements (TEs) in the chromosomes of ***Elaeis guineensis*****.

	**SSR density (/Mb)**	**Gene density (/Mb)**	**TE density (/Mb)**	**Relation coefficient between SSR and TEs**	**Relation coefficient between SSR and genes**
Chromosome 1	723.71	39.30	37.96	−0.51	0.75
Chromosome 2	707.23	36.52	43.29	−0.43	0.65
Chromosome 3	683.53	34.58	39.94	−0.35	0.82
Chromosome 4	659.76	30.01	39.58	−0.34	0.69
Chromosome 5	670.82	36.57	44.15	−0.60	0.84
Chromosome 6	593.98	29.73	45.31	−0.45	0.83
Chromosome 7	676.89	32.47	42.30	−0.44	0.74
Chromosome 8	674.58	35.67	41.22	−0.39	0.85
Chromosome 9	595.82	28.80	42.72	−0.13	0.80
Chromosome 10	724.40	38.76	38.32	−0.29	0.78
Chromosome 11	630.33	32.36	42.14	−0.40	0.86
Chromosome 12	731.01	39.72	41.56	−0.68	0.84
Chromosome 13	701.11	34.87	43.53	−0.69	0.85
Chromosome 14	690.89	38.56	41.26	−0.26	0.71
Chromosome 15	696.67	35.83	44.92	−0.19	0.80
Chromosome 16	735.56	38.00	47.54	−0.39	0.73

### Copy number of these identified microsatellites in genome of *Elaeis guineensis*

A total of 734 509 primer pairs were successfully designed from the flanking sequences of mono- to hexanucleotide microsatellites of *E. guineensis*. Primer pairs could not be designed for the remaining microsatellites, mainly due to lack of sufficient flanking sequence from both sides of the identified microsatellites.

Subsequently, these designed primer pairs were used to *in silico* amplify the assembled genome of *E. guineensis*. A total of 618 762 primer pairs had *in silico* products. Meanwhile, in these primer pairs, 419 958 (67.87%), 45 544 (7.36%), 19 093 (3.09%), and 134 167 (21.68%) could generate 1, 2, 3, and >3 *in silico* PCR products, respectively, from the assembled genomic sequence of *E. guineensis*. A total of 9 687 176 *in silico* fragments could be generated based on these designed primer pairs. Among these *in silico* fragments, 57 989 mono- (accounting for 9.37% of all primer pairs flanking mono-nucleotide motif), 475 326 di- (76.82%), 67 035 tri- (10.83%), 12 561 tetra- (2.14%), 4 272 penta- (0.69%), and 1 579 (0.26%) could produce 139 830, 8 523 025, 784 979, 134 392, 73 686, and 31 264 *in silico* amplification fragments, respectively, from the assembled genome of *E. guineensis*. From these results, 67.87% of primer pairs generate a single *in silico* PCR product, which suggests that these identified microsatellites sequences are only present in one copy in the genome of *E. guineensis*.

Meanwhile, among these designed primer pairs, 228 056 (28.17%) can be mapped onto the assembled chromosomes of *E. guineensis*. A higher proportion of single-copy microsatellites (185 621, 81.39%) was detected in the microsatellites mapped to the assembled chromosomes of *E. guineensis*. Moreover, 14 011 (6.14%), 4 749 (2.08%), and 23 675 (10.38%) primer pairs could produce 2, 3, and >3 *in silico* PCR products, respectively, from the assembled genomic sequence of *E. guineensis*.

### Conservation of microsatellite loci between *Elaeis guineensis* and *Phoenix dactylifera* and the overlap with genic regions

Subsequently, these 618 762 primer pairs were used to *in silico* amplify the assembled genome of *P. dactylifera*. Only 7 265 (0.99%) of these primer pairs designed based on the flanking sequence of microsatellites of *E. guineensis* produced positive e-PCR fragments from the assembled genome of *P. dactylifera* (Table 2). Detailed information for the 7 265 primer pairs is listed in Table S1. For the 7 265 microsatellite markers developed from *E. guineensis*, 4 522 (62.19%), 1 154 (15.91%), 307 (4.22%), and 1 282 (17.68%) could generate 1, 2, 3, and >3 *in silico* PCR products, respectively, from *P. dactylifera*. A total of 65 550 fragments were *in silico* amplified based on these conserved microsatellite primers between *E. guineensis* and *P. dactylifera*. Meanwhile, 458 mono- (accounting for 6.3% of all primer pairs flanking mono-nucleotide motif), 5 443 di- (74.86%), 1 305 tri- (17.95%), 187 tetra- (2.57%), 13 penta- (0.18%), and 12 hexa- (0.17%) primer pairs could produce 458, 56 328, 1 961, 6 961, 40, and 802 *in silico* amplification fragments, respectively, from the assembled genome of *P. dactylifera*.

Among these conserved microsatellite primer pairs, a total of 4 638 primer pairs could be mapped into the assemble chromosomes of *E. guineensis*. A total of 41 710 fragments were *in silico* amplified based on these conserved microsatellite primers between *E. guineensis* and *P. dactylifera*, with an average of 8.99 fragments per primer. A higher proportion of primer pairs (3260, 70.29%) only generated a single *in silico* PCR product in the assembled chromosomes of *E. guineensis*. Moreover, 335 (7.45%), 28 (0.62%), and 24 (0.54%) could generate 1, 2, 3, and >3 *in silico* PCR products, respectively, from the assembled genomic sequence of *P. dactylifera*.

The position of the conserved microsatellites in the assembled genome of *E. guineensis* was analyzed to confirm if they were located within genic or intergenic regions. The analysis results showed that the majority of the conserved microsatellites (71.45%) were located within genic regions, while the others (28.55%) were located within intergenic regions (Figure S3). In different chromosomes of *E. guineensis*, the percentage of conserved microsatellites within genic regions varied from 54.04% in chromosome 11 to 89.41% in chromosome 13. Of these conserved microsatellites in genic regions, 5.5% were located in untranslated regions (UTRs), which may be associated with gene expression and splicing; 28.4% were located in coding regions (CDS), which may be directly associated with amino sequences; and the remaining 66.1% were located in intron regions of genes. The analysis results suggested that these conserved microsatellites are prone to be distributed within genic regions.

### Cross-genera transferability of these conserved microsatellites in the palm species

We chose conserved microsatellite markers that could be detected in both the assembled genomes of *P. dactylifera* and *E. guineensis* for transferability analysis in Palmae species. One hundred and thirty five primer pairs were selected stochastically from the 7 265 conserved microsatellite markers: 93 single-copy microsatellites were selected, 19 microsatellites present in two copies were selected, 5 microsatellites present in three copies were selected, and the remaining microsatellites were present in more than three copies. The 135 primer pairs were used to amplify genomic DNA from 32 palm species. In 106 (78.5% of cases), PCR products could be amplified in the genomic DNA of *E. guineensis*. The remaining 29 primer pairs were excluded from further analysis due to lack of PCR products or due to weak amplification. A total of 328 alleles were detected, with an average of 3.04 alleles per microsatellite marker (Table S2). Meanwhile, these microsatellite markers also showed high transferability to other palm species, ranging from amplification in 17 to 93% of species samples with an average of 73% (Figure S4). These results indicated that these microsatellite loci were conserved in the palm species.

## Discussion

Although the genomic sequences of *E. guineensis* and *P. dactylifera* have been released, there are still no related documents which elucidate and compare the frequency and distribution of microsatellites in the two palm species. To our knowledge, this study is the first report on genome-wide identification and comparison of microsatellite loci based on the assembled genomes of *E. guineensis* and *P. dactylifera*. In previous studies, microsatellites were generally developed based on the released transcriptome data from *E. guineensis* and *P. dactylifera*. Tranbarger et al. (2012) detected 465 microsatellites within 2 652 262 bp of *E. guineensis* EST sequences. Zhao et al. (2012) detected 5981 microsatellites from 28 889 assembled EST sequences from *P. dactylifera*. However, our study was based on the entire assembled genomes of *E. guineensis* and *P. dactylifera*, including non-coding sequences. This allowed a far greater number of microsatellites (814 383 and 371 629 respectively) to be identified compared to previous studies (Tranbarger et al., 2012; Zhao et al., 2012; Aberlenc-Bertossi et al., 2014).

Previously, the frequency of microsatellites was reported to be very similar in different species of the same genera (Shi et al., 2013). In this study, *E. guineensis* and *P. dactylifera* are classified into different genera in the Palmae family. However, the density of microsatellites in the assembled genomic sequences of *E. guineensis* (770.4 microsatellites per Mb) and *P. dactylifera* (733 microsatellites per Mb) is also nearly identical, indicating highly similar genomic structure in the two palm species. Meanwhile, the distribution with respect to the motif type and repeat number of microsatellites in the assembled genomic sequences of the two palm species is also very similar. More specifically, A/T rich motifs are generally dominant/major, while C/G rich motifs are absent/scarce, which is in accordance with reported results by Shi et al. (2013). Microsatellite expansion and variation is thought to result from replication slippage by mismatch during DNA replication (Tautz and Schlotterer, 1994; Klintschar et al., 2004; Forster et al., 2015), which occurs more readily in A/T rich motifs than in C/G rich motifs. Overall, the microsatellite distribution characteristics in the assembled genomics of the two palm species are very similar. This suggests that the two Palmae species have similar genome structure, which may be due to their relatively recent divergence (65 Myr) from a common ancestor (Singh et al., 2013).

In this study, a total of 814 383 and 371 629 mono- to hexanucleotide repeat microsatellites were identified in the assembled genomic sequences of *E. guineensis* and *P. dactylifera*, respectively, with frequencies of 770.4 and 733 microsatellites per Mb or one microsatellite per 1.29 and 1.36. The microsatellite density in the assembled genomic sequences of the two Palmae species is higher than the SSR density in some species (Tautz and Schlotterer, 1994; Klintschar et al., 2004; Song et al., 2015). To our knowledge, this study is the first report on genome-wide identification of microsatellite loci in palm family. Most (99.29 and 98.39% for *E. guineensis* and *P. dactylifera*) of the identified microsatellite loci are novel compared to previous research in these two palm species (Zhao et al., 2012; Xiao et al., 2014). Although further validation may still be required, these newly developed microsatellite loci will be useful for future molecular marker studies in *E. guineensis* and *P. dactylifera*. Possible applications include the construction of high-density linkage maps, QTL fine mapping and genome-wide association mapping.

A great deal of research has been performed to identify genic microsatellites via *in silico* analysis of publicly available unique transcriptome sequences in *E. guineensis* (Tranbarger et al., 2012; Xiao et al., 2014). However, due to the lack of genomic sequences, the distribution and physical position of these publicly available microsatellite markers is still unclear, which has a negative effect on their effective utilization. In this study, we describe the genome distribution and the accurate physical position of these newly developed microsatellite markers based on the mapped sequence scaffolds of *E. guineensis*. The high-density microsatellite markers with accurate physical position could be very useful for rapid selection of trait-associated markers and subsequent application of molecular breeding in *E. guineensis*. Previous studies have showed that the distribution of microsatellites is preferentially associated with non-repetitive DNA/gene sequences in plant genomes (Morgante et al., 2002; Gemayel et al., 2010). In our study, the genomic distribution of microsatellites was also positively correlated to that of genes but negatively correlated to that of TEs. The high accordance between the genomic distribution of microsatellites and genes may suggest a putative role of microsatellite in regulating gene function (Li et al., 2002, 2004; Gemayel et al., 2010). Some microsatellite markers could also be used as functional markers to tag genes and for molecular breeding.

Microsatellite markers obtained from one plant species are seldom used directly in populations of closely related species. In order to screen cross-genera transferability of microsatellite markers, primer pairs flanking microsatellites from one plant species are generally used to amplify DNA from other species in the same genera (Chagne et al., 2004; Guo et al., 2006). This method has comparatively low efficiency. Normally, only a few cross-species transferable microsatellite markers can be obtained through the selection of a large number of primer pairs. This study was conducted to select cross-genera-transferable microsatellite markers in the Palmae family. In order to increase the selection efficiency, a large number of conserved microsatellites were first identified between *E. guineensis* and *P. dactylifera*. Most of these conserved microsatellites identified in the present study were also validated to have high transferability ratio in palm species in the study. In conclusion, the conserved microsatellites identified in the study will provide some genetic resources for the assessment of genetic diversity, the analysis of population structure, and conservation of palm species, especially for endangered species.

Electronic PCR (e-PCR) is a useful tool for simulating the process of Polymerase Chain Reaction (PCR) and for detecting potential amplification products. In the present study, validating transferability for 618 762 primer pairs would be prohibitively time-consuming. Electronic PCR (e-PCR) simplified the process. The selection of parameters is important for the reliability of electronic PCR (e-PCR). The default parameters of the software are 2 bp mismatch, 1 bp gap, and 500–1000 bp product size. In the present study, in order to increase the reliability of the electronic PCR results, more stringent parameters were applied: a 1 bp mismatch and 0 bp gap were allowed in order to decrease the chance of false positives, although this may also increase the false negative rate.

Location of microsatellites in non-coding regions allows easier accumulation of mutations over the generations, and gives rise to increased variability which can be used for DNA markers. However, other microsatellites may be located in the UTR of mRNA, promoter regions of expressed genes, or even directly in CDS of genes. In such cases, microsatellite mutation may lead to phenotypic changes. Microsatellite variation in CDS can lead directly to functional protein changes, while microsatellite variation occurring in 5′ UTR (5′-UTRs) may affect transcription and translation, and microsatellite variation in 3′-UTRs may affect splicing. Therefore, these microsatellites associated with biological functions due to their location may be more highly conserved between different genotypes and even between different species, as most novel mutations are detrimental. In this study, some microsatellites were found to be conserved between palm species, and the position of these conserved microsatellites in the genome of *E. guineensis* was also analyzed. The majority of the conserved microsatellites (71.45%) were located within genic regions, while the remainder (28.55%) were located within intergenic regions. It is possible that some conserved microsatellites in intergenic regions were located on promoter regions of genes, such that mutation of these microsatellites may lead to a change in gene expression. Mutation of the 71.45% of conserved microsatellites located in genic regions may directly lead to changes in amino sequence, splicing, and gene expression. In summary, microsatellites associated with biological function due to their genomic location in genic regions may be more conserved between different genotypes and even species, and hence more transferable to other species. Therefore, selection of genic microsatellites may be a useful strategy for identifying high proportions of markers transferable between species.

## Conclusions

In the last two decades, advances in next-generation sequencing technology have facilitated the development of molecular markers. In this study, genome-wide identification of microsatellites was conducted based on the assembled genomic sequences of *E. guineensis* and *P. dactylifera*. A high density of microsatellites was identified in the two species, which will provide a useful tool for research into genetic diversity, gene mapping and molecular breeding in these species.

It has been previously documented that some microsatellites could be transferable between species or even between genera (Chagne et al., 2004; Mnejja et al., 2005; Guo et al., 2006; Pandey et al., 2013), In this study, we tried to develop a large number of conserved microsatellites which can be used for genetic analyses in different palm species. A total of 7 265 conserved microsatellite markers were identified between *E. guineensis* and *P. dactylifera*. In the future, these identified conserved SSR markers may be regarded as useful genetic resources for diversity conservation in different species in the Palmae family, especially in endangered species.

## Author contributors

YX and WX did the DNA extract and subsequently PCR amplification, participated in the design of the study, performed the statistical analysis and drafted the manuscript. JM did the extraction of DNAs and PCR amplification. AM critically revised the manuscript. XL, HF, ZM, and MP participated in the design of the study. All authors read the final manuscript and have given final approval of the version to be published.

## Funding

This work was supported by the Natural Science Foundation of China (No.31301358), the Scientific and Technological Cooperation Projects of Hainan province (No.SQ2015GJXM0076), National Nonprofit Institute Research Grant of CATAS-ITBB (No.1630052015050), and The Major Technology Project of Hainan (ZDZX2013023-1).

### Conflict of interest statement

The authors declare that the research was conducted in the absence of any commercial or financial relationships that could be construed as a potential conflict of interest. The reviewer CZ declared a shared affiliation, though no other collaboration, with several of the authors ZM, MP to the handling Editor, who ensured that the process nevertheless met the standards of a fair and objective review.
