# Supplementary material for: Genome-Wide Identification and Transferability of Microsatellite Markers between Palmae Species
Source: Front Plant Sci. 2016 Oct 25;7:1578. doi: 10.3389/fpls.2016.01578 (PMC5078683; doi:10.3389/fpls.2016.01578)
Supplement: Supplementary file 3 [file Image1.PDF]

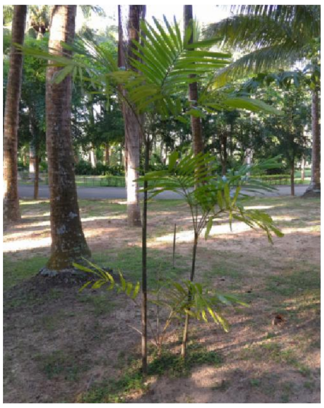
Chrysalldocarpus lutescens

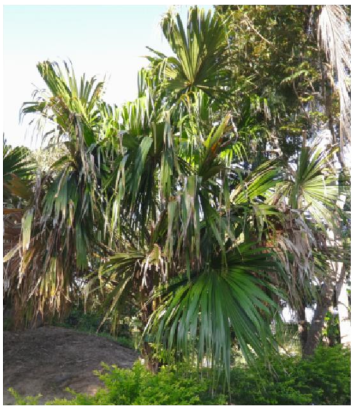
Livistona australis

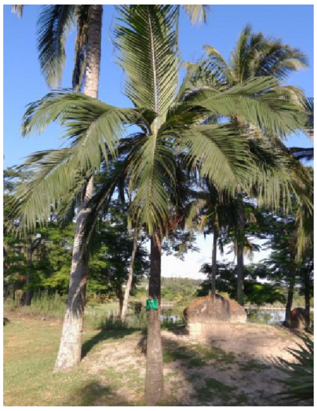
Dictyosperma album

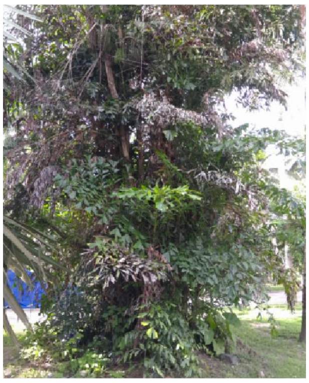
Caryota mitis

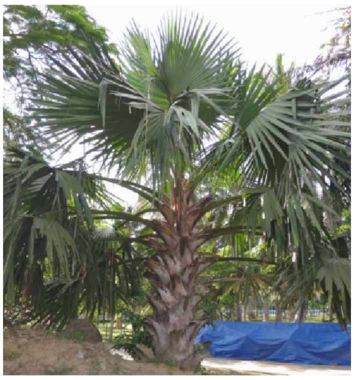
Corypha umbraculifea

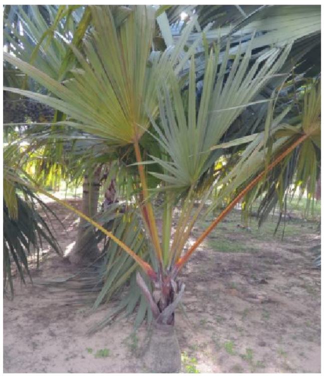
Latanic lontaroides

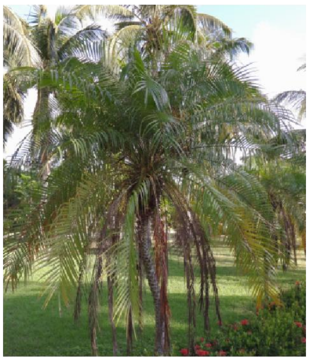
Phoenix loureirii

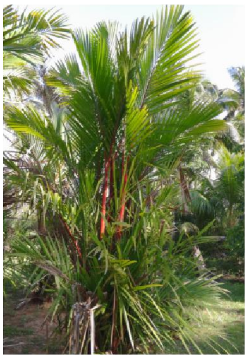
Cyrtostachys renda

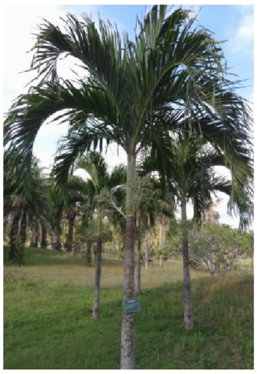
Veitchia merrillii

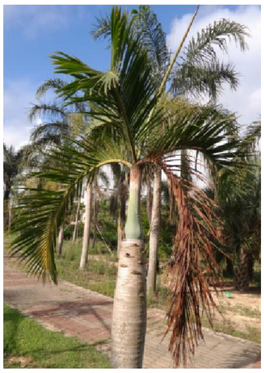
Hyophorbe verschaffeltii

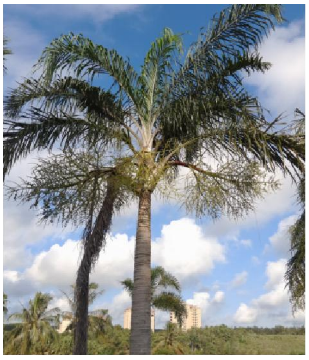

Chrysalidocarpus lucubensis

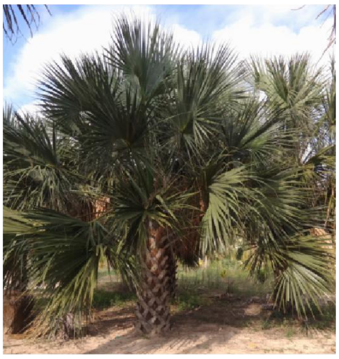

Sabal palmetto

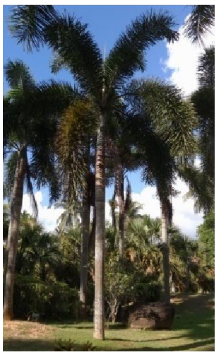

Wodyetia bifurcate

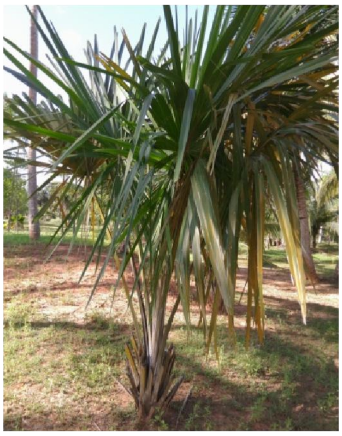

Trachycarpus nana

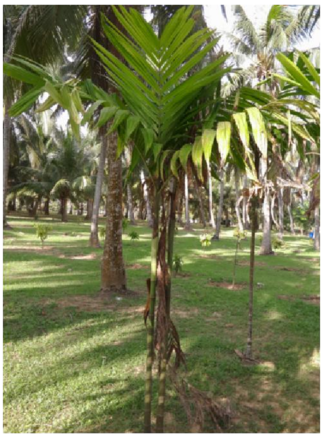

Areca triandra

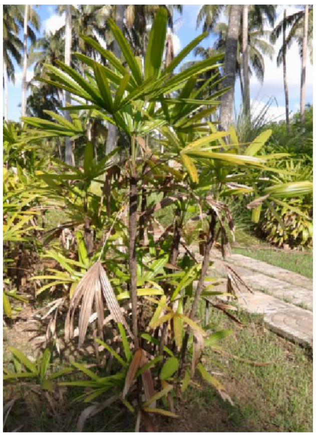

Rhapis excels

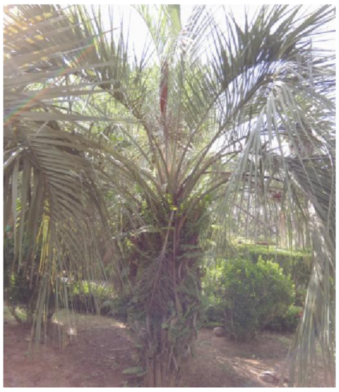

Butia capitata

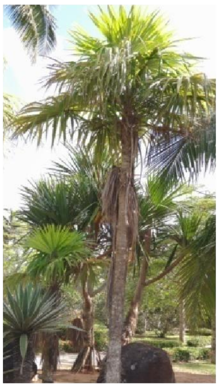

Cryosophila albida

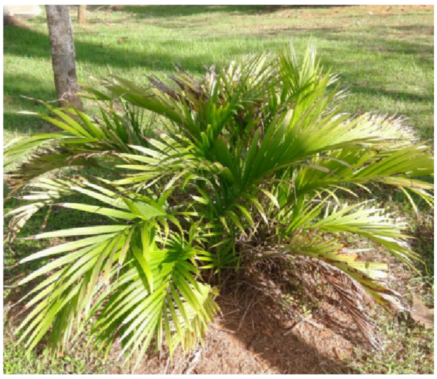

Chamaedorea Elegans

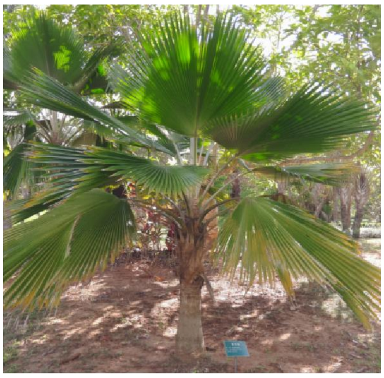

Pritchardia pacifica

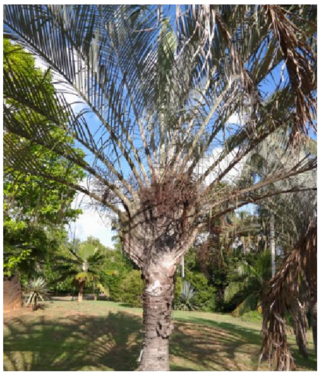

Dypsis decaryi

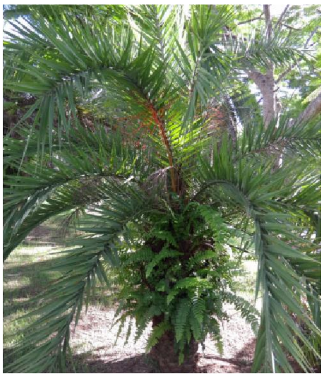

Phoenix robusta

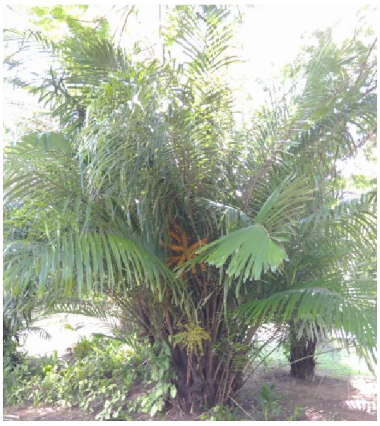

Arenga engieri

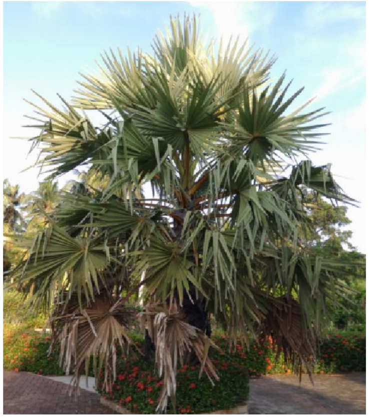

Borassus flabellifer

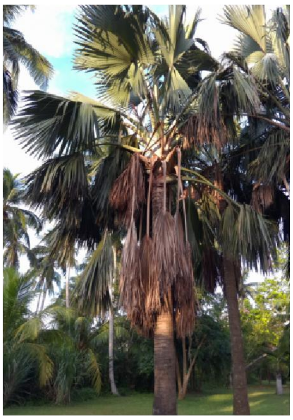

Bismarckia Hildebr

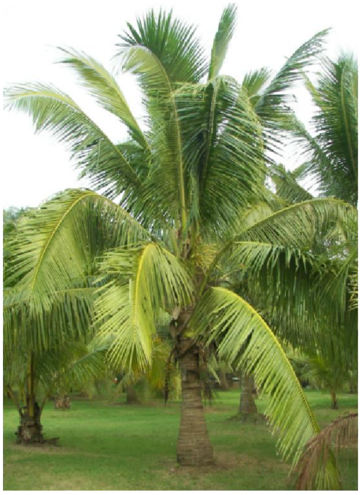

Cocos nucifera

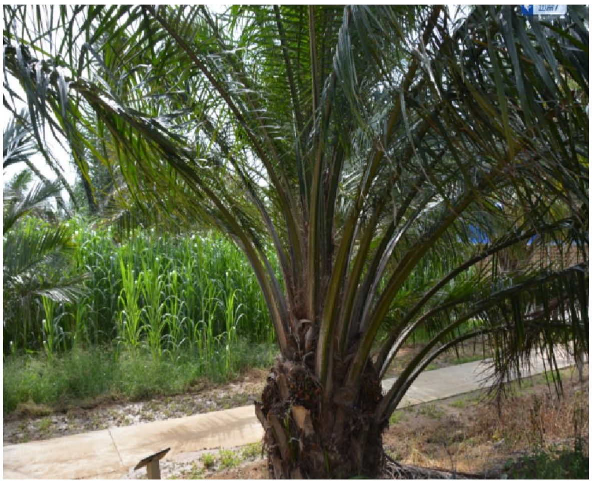

Elaeis guineensis

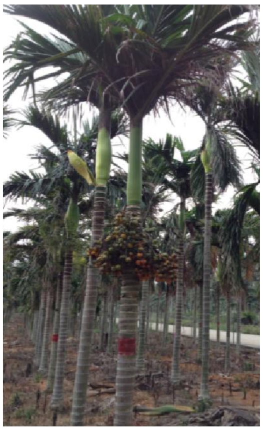

Areca catechu

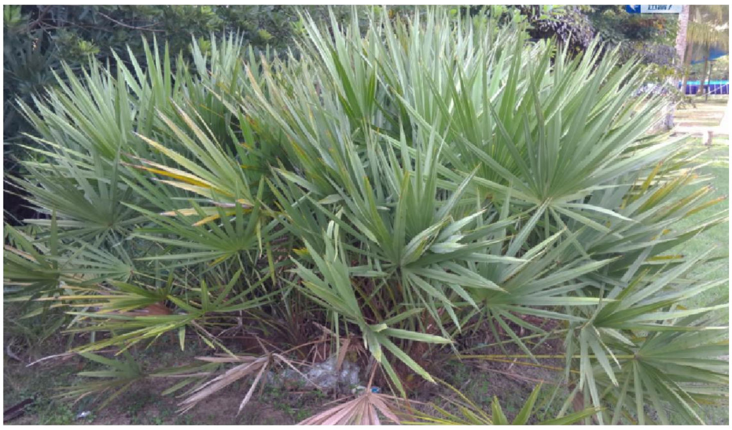

Chamaerops ritchieana

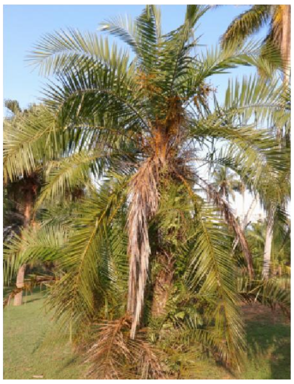

Iraq candy date

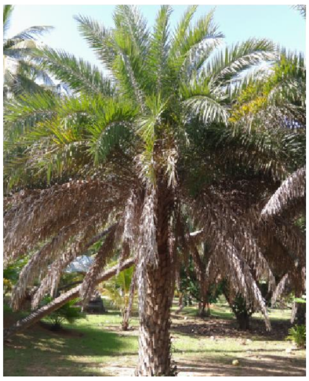

Phoenix dactylitfera

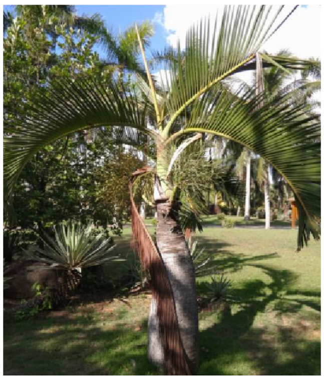

Hyophorbe lagenicaulis

Fig. S1 Morphological characteristics of 32 palmae species used in the study.
