# Supplementary material for: Genome-Wide Identification and Transferability of Microsatellite Markers between Palmae Species
Source: Front Plant Sci. 2016 Oct 25;7:1578. doi: 10.3389/fpls.2016.01578 (PMC5078683; doi:10.3389/fpls.2016.01578)
Supplement: Supplementary file 4 [file Image2.PDF]

Chromosome 5

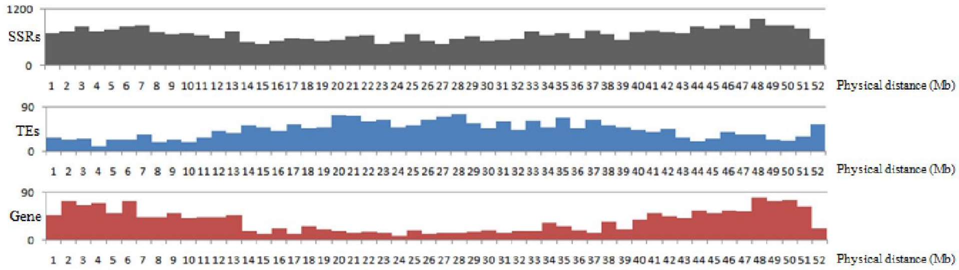

Chromosome 6

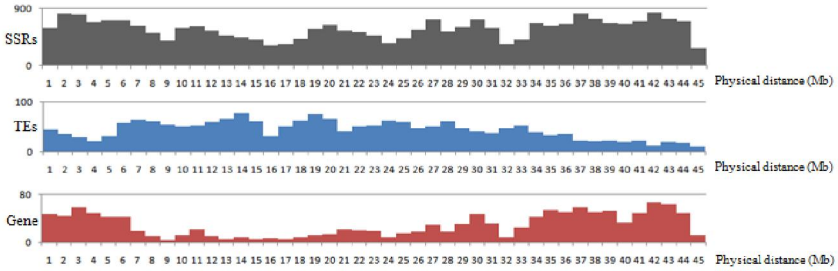

Chromosome 7

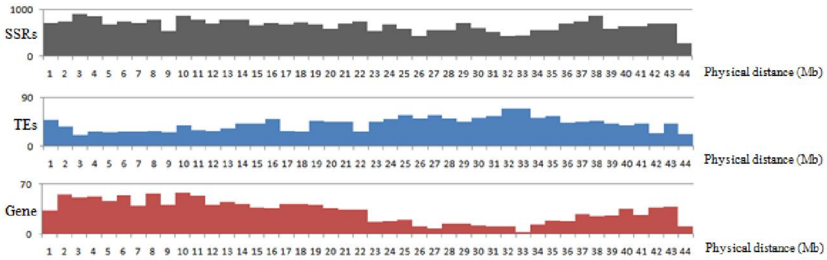

Chromosome 8

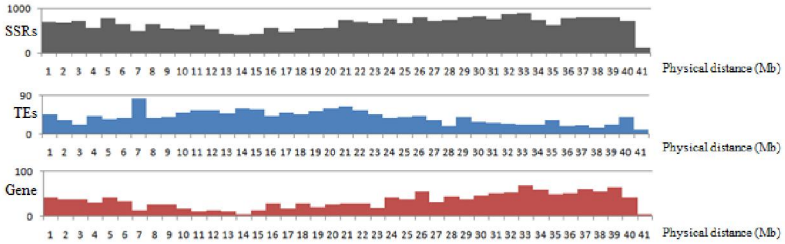

Chromosome 9

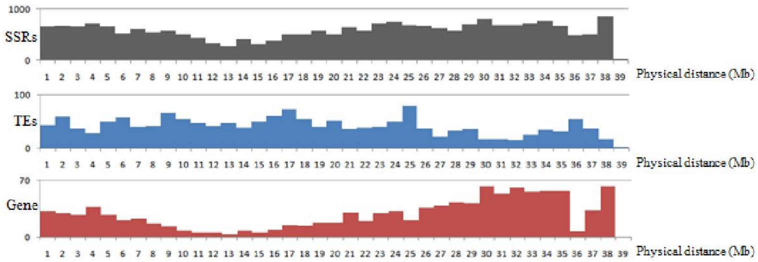

Chromosome 10

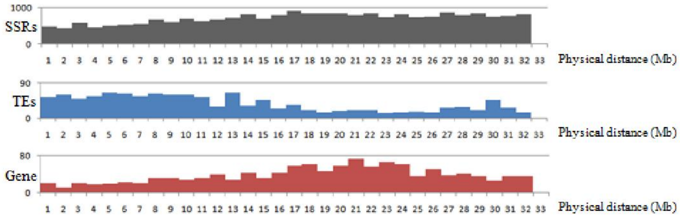

Chromosome 11

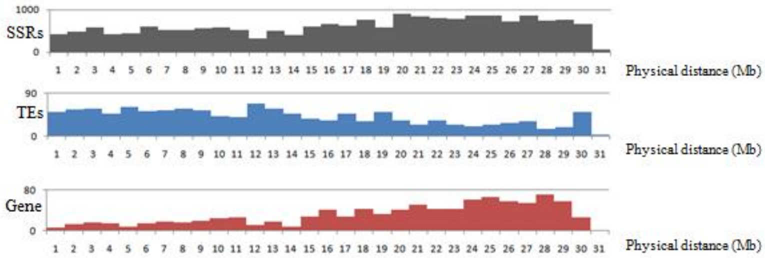

Chromosome 12

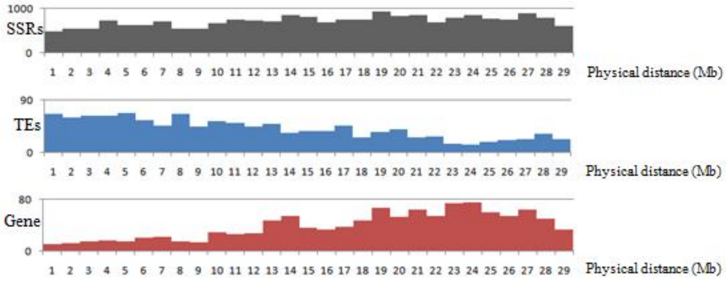

Chromosome 13

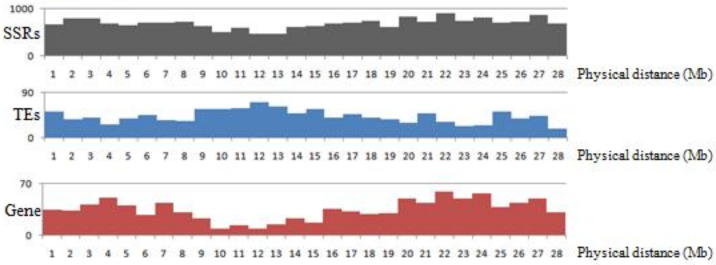

Chromosome 14

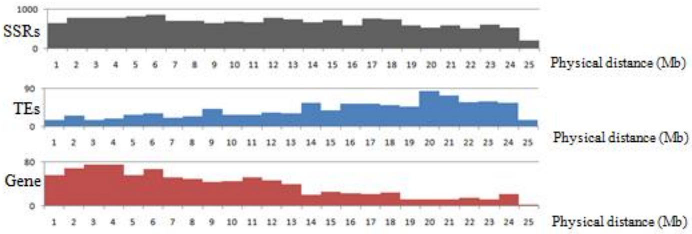

Chromosome 15

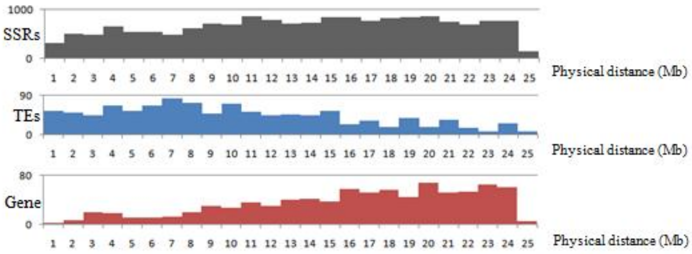

Chromosome 16

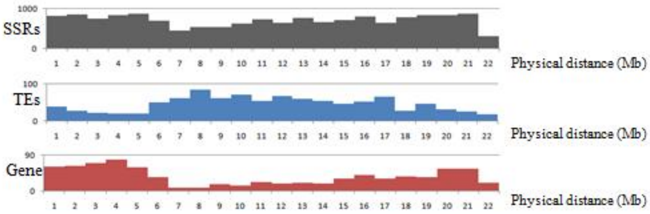

Fig. S2 Distribution and density of microsatellite as well as genes and TEs in the assembled genome of *Elaeis guineensis* (from chromosome 5 to chromosome 16). The black, blue and red bar represented the number of microsatellite, TEs and genes, respectively. The horizontal axis showed the physical distance of the assembled chromosomes.
