# Supplementary material for: Genome-Wide Identification and Transferability of Microsatellite Markers between Palmae Species
Source: Front Plant Sci. 2016 Oct 25;7:1578. doi: 10.3389/fpls.2016.01578 (PMC5078683; doi:10.3389/fpls.2016.01578)
Supplement: Supplementary file 5 [file Image3.PDF]

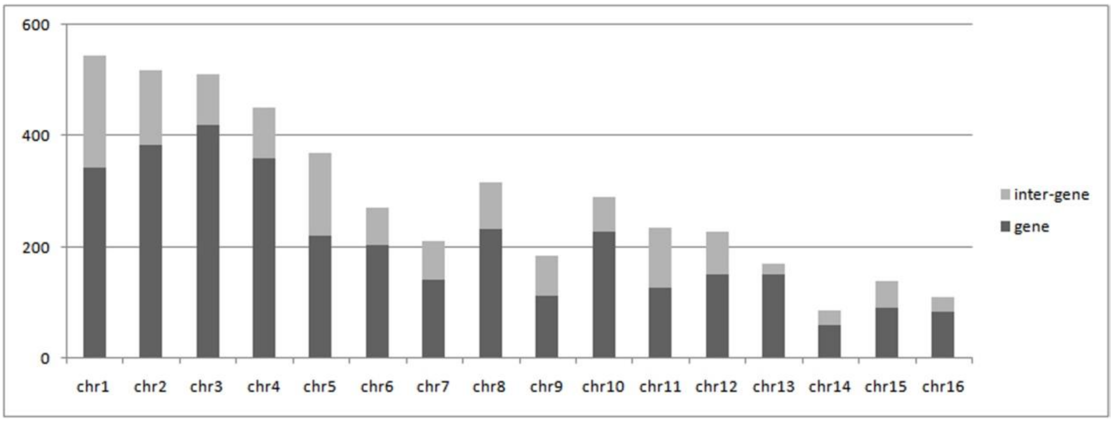

Fig. S3 Number of conserved microsatellites and their distribution between genic and intergenic region.
