# Supplementary material for: Genome-Wide Identification and Transferability of Microsatellite Markers between Palmae Species
Source: Front Plant Sci. 2016 Oct 25;7:1578. doi: 10.3389/fpls.2016.01578 (PMC5078683; doi:10.3389/fpls.2016.01578)
Supplement: Supplementary file 6 [file Image4.PDF]

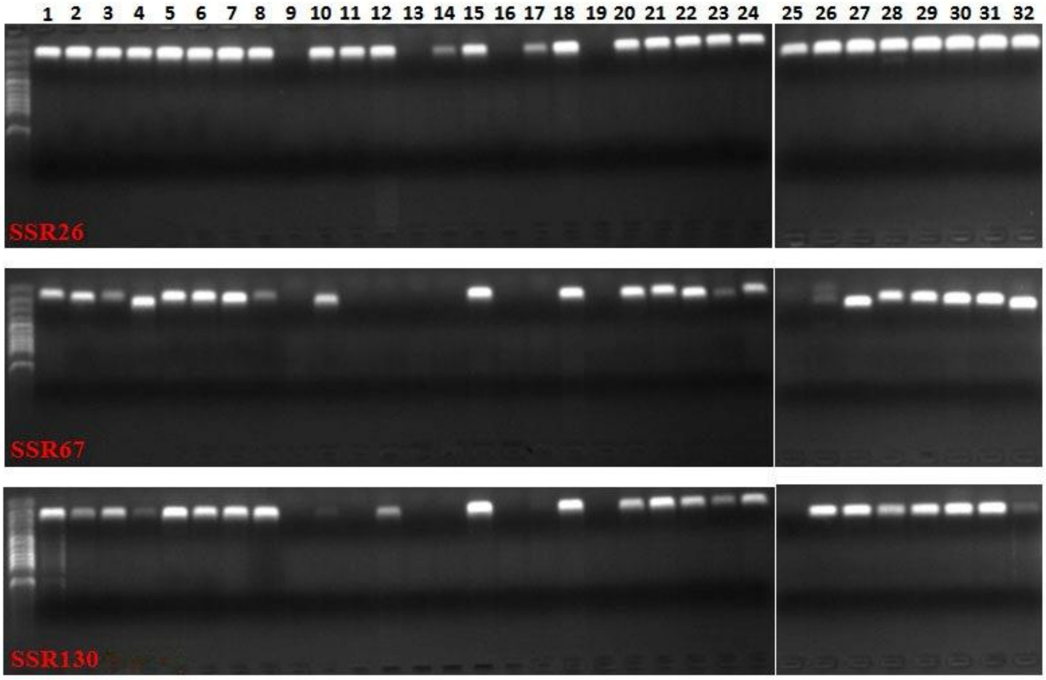

Fig. S4 PCR products of conserved SSR markers across the genomic DNA of 32 palm species. Arabic numbers 1 to 32 represent different palm species: 1. *Chrysalldocarpuslutescens*; 2. *Livistonaaustralis*. 3. *Dictyosperma Album*. 4. *Cary mitis*. 5. *Coryphaumbraculifea*. 6. *Lataniclontaroides*. 7. *Phoenix loureirii*. 8. *Cyrtostachysrenda*. 9. *Veitchiamerrillii*. 10. *Hyophorbeverschaffeltii*. 11. *Chrysalidocarpuslucubensis*. 12. *Sabal palmetto*. 13. *Wodyetiabifurcate*. 14.*Trachycarpus nana*. 15. *Areca triandra*. 16. *Rhapis excels*. 17. *Butiacapitata*. 18. *Cryosophilaalbida*. 19. *ChamaedoreaElegans*. 20. *Pritchardiapacifica*. 21. *Dypsisdecaryi*. 22. *Phoenix robusta*. 23. *Arengaengieri*. 24. *Borassusflabellifer*. 25. *BismarckiaHildebr*. 26. *Cocosnucifera*. 27. *Elaeis guineensis*. 28. *Semen Arecae*. 29.*Chamaeropsritchieana* 30. Iraq candy date. 31. *Phoenix dactylifera*. 32. *Hyophorbelagenicaulis*.
